# Migrant adolescents’ behavioral problems compared to host adolescents and adolescents in their region of origin: a longitudinal study

**DOI:** 10.1186/s12888-020-02872-x

**Published:** 2020-09-29

**Authors:** Jian-Qun Fang, Yan-rong Wang, Yun-Yun Du, Guo-Li Yan, Fu-Li Ma, Yan-Qiu Liu, Wen-Xi Sun, Shi-Qi Chen, Li-Ping Feng, Jia Wei, Hao Liu, Jing Hu, Zhao-Xia Zhang

**Affiliations:** 1 Mental Health Center, The General Hospital of Ningxia Medical University, No. 804 South Shengli Street, Yinchuan, 750004 Ningxia China; 2 School of Nursing, Shandong University, No.44 Wenhua Xi Road, Jinan, 250012 China; 3 Ningxia Medical University, No.1106 South Shengli Street, Yinchuan, 750004 China; 4 TianJin Anding Hospital, Tianjin Mental Health Institute, Tianjin, 300222 China; 5 Sozhou Guangji Hospital, NO.11, Guangqian Road, Suzhou, 215133 District of Suzhou China

**Keywords:** Ecological migration, Adolescents, Behavioral problems, Personality, Classroom environment

## Abstract

**Background:**

Since the 1990s, families from the ecologically hostile mountainous southern areas of Ningxia Province, China, have been migrating to the northern areas of the province. This study compared the prevalence of behavioral problems among migrant adolescents to those among host adolescents (adolescents from the northern areas) and adolescents in the region of origin (adolescents from the southern areas), to determine whether ecological migration is related to adolescent behavioral problems, and possible changes in such problems over time.

**Methods:**

We used the Children and Adolescents Ecological Migration Survey on Mental Health, administered to 4805 students aged 12–16 years and their parents between 2012 and 2014 (W1), of whom 1753 students and their parents completed the follow-up between 2014 and 2017 (W2). Parents answered questions related to adolescent behavioral problems, main source of family income, parents’ desire to reverse migrate, improved standard of living, and parents’ educational attainment, while children completed the Eysenck Personality Questionnaire and a classroom environment questionnaire.

**Results:**

The prevalence of behavioral problems among the migrant adolescents (28.04%) was significantly higher than among host adolescents (21.59%) or adolescents in the region of origin (24.37%; *p* < 0.001) at W1. After adjusting for gender and age, parents’ work outside the home was the main source of family income (OR = 1.42, 95% CI = 1.13–1.78), and adolescents’ learning burden (OR = 1.04, 95% CI = 1.01–1.06) in school negatively influenced behavioral problems. Strong student-teacher relationships (OR = 0.97,95% CI = 0.94–0.99) and parents who had no intention to move back to the original residence (OR = 0.70, 95% CI = 0.52–0.94) exerted a protective effect at W1; at W2, a protective effect was associated with improved living conditions (OR = 0.39–0.55, 95% CI = 0.25–0.84). The extent of behavioral problems among migrant adolescents significantly decreased after two years.

**Conclusion:**

Ecological migration will increase children’s behavioral problems in the early stage, with various factors influencing the extent of these problems.

## Background

Population migration is an important phenomenon in the development of human societies. It is now an indispensable, inevitable, and potentially beneficial component of the economic and social life of every country and region [1]. The most common form of migration is voluntary migration from a developing country to a developed country [2]. However, environmental degradation (e.g., desertification and drought) can also force people to move elsewhere in order to maintain short-term or long-term survival, which is called ecological migration [3]. Compared with traditional migrants, ecological migrants in Ningxia have a unique history and cultural background [3]. Ningxia Province is located in western China, where the natural conditions in the north and south are extremely different. The northern area benefits from the irrigation from the Yellow River, and the land is fertile. The southern mountainous areas are located in the rugged Loess Plateau region, receive scant rainfall, and have poorly nourished land. The residents’ survival has been threatened by a lack of natural resources, serious environmental degradation, and the poverty of its undeveloped rural areas. In the 1990s, the Chinese government developed a “Migrant Comprehensive Relocation Project” to coordinate voluntary relocation of residents in the southern mountainous areas to the northern plains region of Ningxia. With the support of the Ningxia government (including family-based housing, community-based road repair, and public transport development), people living in adverse ecological areas and poor counties (districts) have relocated with family to better living conditions. As of 2011, approximately 30% of the total population in those areas was involved with the Ningxia emigration project [4].

The post-immigration experience can have implications for immigrants’ mental health [5], especially among youth [6–9]. For example, a recent study showed that immigrant youth are more likely to have poorer mental health outcomes than are non-immigrants [10–13]. Mijnke et al. [14] studied the impact of immigration on adolescents’ emotional and behavioral problems and found higher rates of emotional and behavioral issues among immigrant youth. Buchmuller et al. [15] found that refugee parents reported more mental health concerns in relation to their children, especially regarding internalizing difficulties. In addition, researchers also surmised that immigrants’ situations may differ based on their specific ethnic backgrounds and the specific reasons for immigration among immigrant youth [16]. However, other studies found that migration only had a small impact when predicting the development of mental health problems in immigrant adolescents [17].

Theories of the impact of migration on emotional and behavioral problems have suggested both a risk and a resilience perspective. The risk perspective focuses on the potential stress generated by migration, such as through the loss of family and friends, the environment, and the need to adapt to a new cultural environment [18, 19]. Generally, ecological emigration might require relocation to an alien environment far from one’s neighborhood and family, and adaptation to a different way of life. Although the government provides these emigrants with the basic resources they need for daily life and plots for farming, the migrants inevitably experience many stressors that might influence their mental health because of the major changes in circumstance they experience at their destinations. Additionally, adolescence has been identified as a period of susceptibility to mental health issues, such as behavioral problems, that can have profound short-term and long-term impacts on overall health and well-being [20]. Adolescents who relocate with their parents are even more susceptible to behavioral problems. The process of acculturation and the economic hardship usually experienced by migrant children increases their risk of developing behavioral problems [21]; however, many children of migrants have proven to be extremely resilient, despite risk and adversity [22]. The resilience perspective claims that the types and extent of adolescent immigrants’ emotional and behavioral problems are similar to or less than those of their peers, which is referred to as the “immigration paradox” [23]. This paradox has been explained by the combined influences of the adolescents’ sense of family ties, social support, cultural beliefs and practices, and their relatively low rates of substance abuse as a buffer that protects them from emotional and behavioral problems such as mood, anxiety, and personality disorders [24–26].

The results of studies on the behavioral problems of immigrant children are conflicting. Some studies found that immigrant children have greater behavioral problems than do native-born children [27], whereas other studies have found the reverse [28]. It is not clear whether the effects of ecological migration on adolescent behavioral problems are consistent with the results of general migration. To our knowledge, no longitudinal studies have been conducted to comprehensively examine the behavioral problems of migrant adolescents compared with those of adolescents in their host region and those who remained in their region of origin. The present study assesses the current prevalence of behavioral problems among migrant adolescents. First, migrant adolescents were compared with host adolescents (i.e., adolescents who have been living in the migration destination and have not experienced family relocation in the previous 2 years) and adolescents in the region of origin (i.e., adolescents who remained in their source region and have not experienced family relocation in the previous 2 years). Second, changes in behavioral problems over time were explored by comparing the differences between the first stage (6 months to 2 years after migrating to the northern resettlement area) and the second stage (from 2 years to 4 years).

To date, little empirical evidence exists by which to conduct comprehensive analysis at multiple levels of factors with respect to the behavioral problems of migrant adolescents. McLeroy’s ecological perspective may be particularly applicable to such an analysis. McLeroy [29] proposed that behavior is determined by multiple levels of factors, including intrapersonal, interpersonal, organizational, community, and public policy. Based on McLeroy’s theory, we selected and examined four levels of factors—individual, family, school environment, and ecological migration—that potentially affect behavioral problems in adolescent ecological migrants. This approach provides insight into the complex and multilevel aspects of the relationships between migration-related factors and behavioral problems among adolescent ecological migrants.

In brief, we explored the following questions: Does ecological migration affect behavioral problems in adolescents? Does the prevalence of behavioral problems in migrant adolescents change over time? What are the risk and protective factors that influence behavioral problems in migrant adolescents?

## Methods

### Design and participants

The data used for this study were from Wave I (W1, 2012–2014) and Wave II (W2, 2014–2017) of the Children and Adolescents Ecological Migration Survey on Mental Health in Ningxia. A multi-stage sampling method was used in the study, in which 144 classes in 18 schools were randomly selected from among 9 counties and cities in the northern area that received migrants, and 72 classes in 18 schools were randomly selected from among 9 counties and cities in the mountainous southern area.

Criteria for inclusion in the group of children and adolescents who had experienced ecological migration were: students aged 6–16 years belonging to families that the government had moved from the mountainous area of southern Ningxia to the northern resettlement area, and who had been living in the resettlement area for a period from 6 months to 2 years. Criteria for inclusion in the group of host children and adolescents were: students aged 6–16 years who lived in the northern area and had not experienced family relocation in the prior 2 years. Inclusion criteria for the group of adolescents in the region of origin were: primary- and middle-school students, aged 6–16 years, who lived in the mountainous southern area, and had not experienced family relocation in the prior 2 years. Exclusion criteria for each group were: the presence of physical disease, intellectual disability, or severe mental disorder. Severe mental disorders were determined based on parent report of whether their child had any clinically diagnosed mental disorders. This manuscript primarily considered the prevalence of behavioral problems and related factors among adolescents aged 12–16 years, and selected cases that met the inclusion criteria from the full database.

**Fig. 1:**
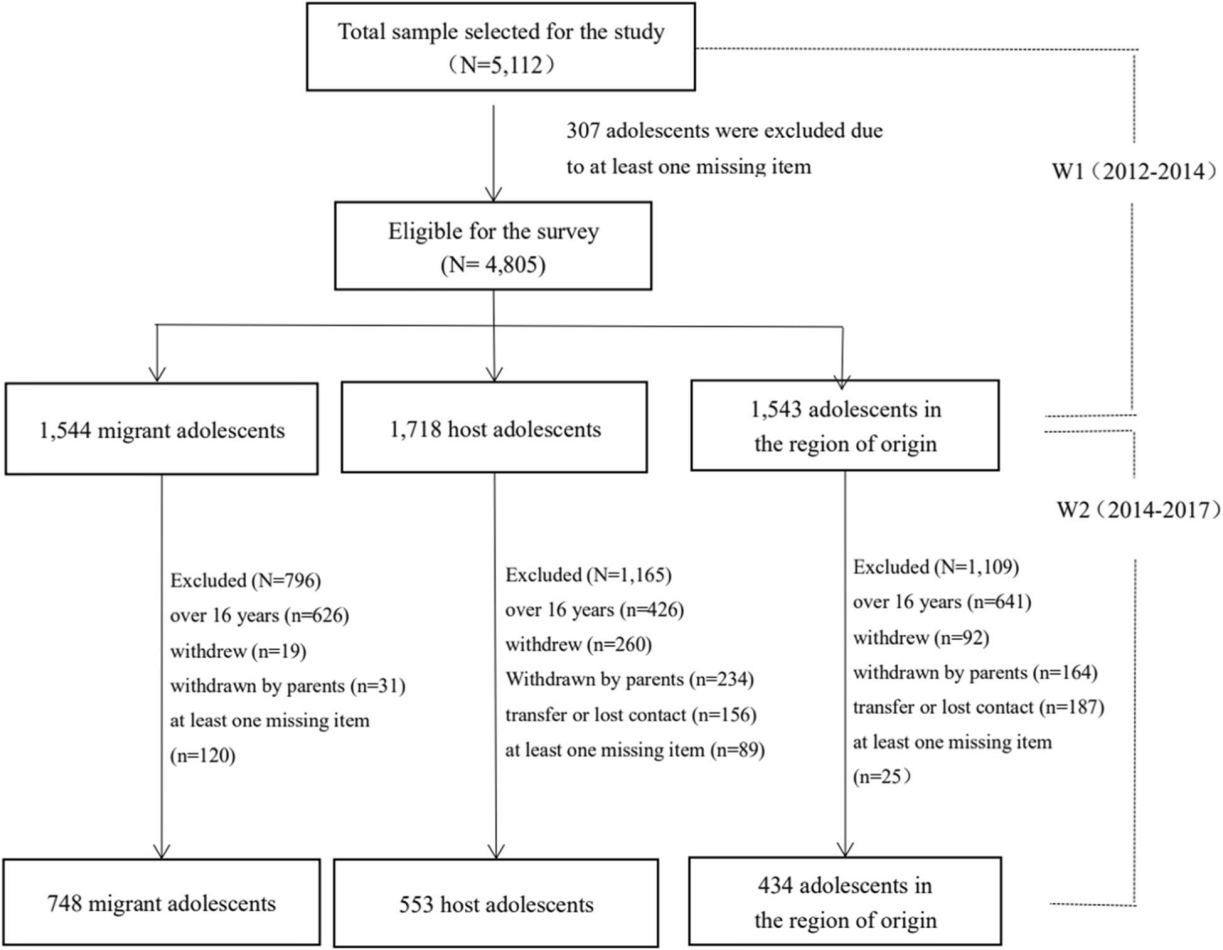
Participation flowchart

Parents answered questions related to adolescent behavioral problems, main source of family income, parents’ desire to reverse migrate, improved standard of living, and parents’ educational attainment, while children completed the Eysenck Personality Questionnaire (EPQ) and classroom environment questionnaire. In total, 5112 students and their parents completed the survey at W1, of whom a final sample of 4805 respondents was analyzed after excluding responses with missing data. The final sample consisted of 1544 migrant adolescents, 1718 host adolescents, and 1543 adolescents in the region of origin. From 2014 to 2017 (W2), a follow-up survey was conducted based on the W1 survey, which assessed 1735 respondents: 748 migrant adolescents, 553 host adolescents, and 434 adolescents in the region of origin. Figure 1 presents the participation flowchart.

The protocol for this study was approved by the Ethics Committee of the General Hospital of Ningxia Medical University. As all participants were under 16 years of age, written informed consent was obtained from their parent or guardian, who were informed of the study objectives. The parents/guardians then completed the self-report questionnaires.

### Variables and measurement

#### Child behavior checklist (CBCL)

The CBCL has 113 items across 8 dimensions: (1) withdrawal, (2) somatic complaints, (3) anxiety/depression, (4) social problems, (5) cognitive problems, (6) attention problems, (7) delinquent behaviors, and (8) aggressive behaviors. The CBCL is a parent-report scale that measures adolescents’ behavior during the past three to 6 months via a four-point (0–3) Likert scale [30]. Cronbach’s alpha was 0.956 in the current sample.

#### Personality

The Eysenck Personality Questionnaire (EPQ) was used to measure the adolescents’ personality traits as (1) extraversion (“E”: manifested in outgoing, energetic behavior), (2) psychoticism (“P”: characterized by aggressiveness and interpersonal hostility), or (3) neuroticism (“N”: typified by emotional instability) [31].

#### Parents’ educational attainment

The parents’ educational level was assessed through responses to the question: “What educational background do you and your spouse have?” The data were categorized as 1 = both have less than primary school education or 2 = at least one parent completed junior high school or above.

#### Main source of family income

The family’s livelihood was assessed using responses to the question: “What is your main source of family income?” The data were grouped into two categories: 1 = farming and 2 = working outside the home.

#### Classroom environment

The adolescents’ classroom environment was assessed using the My Class Inventory Questionnaire (MCI) [32]. The questionnaire has 38 items across five dimensions regarding the classroom environment: (1) teacher-student relationship, (2) classmate relationships, (3) order and discipline, (4) peer competition, and (5) learning difficulty.

#### Parents’ desire to reverse migrate

The intensity of the parents’ desire to move back to their place of origin was assessed by asking the question: “Do you or your spouse have a strong desire to move back to your place of origin?” There were three response categories: 1 = no, 2 = occasionally, and 3 = often.

#### Improved living conditions

To learn about the immigrants’ levels of satisfaction with their new living environments, we asked the following question: “Do you think your living conditions are better than before you immigrated here?” The response options were rated as 1 = no, 2 = somewhat, and 3 = completely.

### Data analysis

Epidata 3.02 was used for data entry, and SPSS 17.0 software was used for the statistical analysis. Frequencies/percentages and means/standard deviations are presented to describe the distributions of participants based on their sociodemographic characteristics. Differences in the sociodemographic characteristics among the three groups were examined using one-way analysis of variance (ANOVA) for the continuous variables and Chi-square analysis for contingency tests for the categorical variables. Binary logistic regression models were developed to estimate the odds ratios (ORs) regarding the risk and protective factors that predict behavioral problems across the three groups of adolescents, adjusting for the effects of the sociodemographic variables. All reported *p*-values are two-tailed; the level of statistical significance was set at *p* < 0.05.

## Results

### Sample characteristics

Table 1 shows that respondents’ sociodemographic characteristics varied across the three groups of adolescents (i.e., migrant adolescents, host adolescents, and adolescents in the region of origin). The migrant adolescents were significantly older on average than were the other two groups (*p* < 0.001), and there were significantly more girls in the host adolescent group than in the other groups (*p* < 0.05). Additionally, the parents of the migrant adolescent group reported significantly lower education and working outside the home as the main source of family income than did the parents of the other two groups (*p* < 0.001).

**Table 1:** Descriptive statistics of the participants

	W1					W2				
	Migrant Adolescents	Host Adolescents	Adolescents in the region of origin			Migrant Adolescents	Host Adolescents	Adolescents in the region of origin		
Variable	*N* = 1544(%)	*N* = 1718(%)	*N* = 1543(%)	*F*/χ^2^	*p*	*N* = 748(%)	*N* = 553(%)	*N* = 434(%)	*F*/χ^2^	*p*
Age, mean (SD)	14.44 ± 1.14	13.56 ± 1.33	13.98 ± 1.47	179.30	< 0.001	15.49 ± 0.78	14.71 ± 0.72	14.97 ± 0.73	180.38	< 0.001
Gender										
Male	744 (48.19)	839 (48.84)	688 (44.59)	6.66	0.036	370 (49.47)	266 (48.10)	186 (42.86)	4.98	0.083
Female	800 (51.81)	879 (51.16)	855 (55.41)			378 (50.53)	287 (51.90)	248 (57.14)		
Parents’ highest education										
primary school	836 (54.15)	582 (33.88)	700 (45.37)	137.05	< 0.001	363 (48.53)	244 (44.12)	190 (43.78)	3.57	0.168
Middle school and above	708 (45.85)	1136 (66.12)	843 (54.63)			385 (51.47)	309 (55.88)	244 (56.22)		
Family income sources										
Farming	650 (42.10)	896 (52.15)	974 (63.12)	136.88	< 0.001	128 (17.11)	261 (47.20)	337 (77.65)	423.17	< 0.001
Working outside the home	894 (57.90)	822 (47.85)	569 (36.88)			620 (82.89)	292 (52.80)	97 (22.35)		

### Prevalence of adolescent behavioral problems

**Fig. 2:**
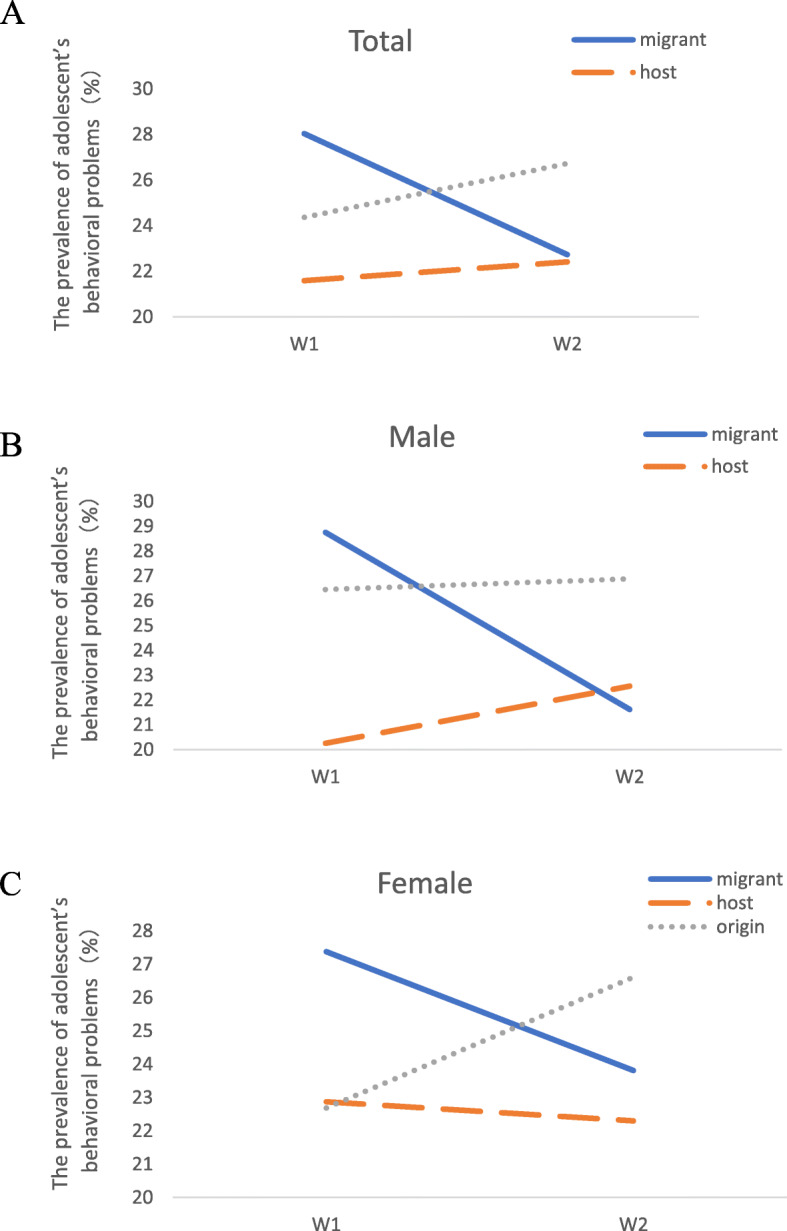
The prevalence of adolescents’ behavioral problems at different stages

A cut-off CBCL score of 36 points indicates behavioral problems (the total CBCL score mean for boys aged 12–16 years was 35.5 points, and the total CBCL score mean for girls aged 12–16 years was 35.8 points) [33]. At W1, the migrant adolescent group had the highest prevalence of behavioral problems (28.04%) compared to host adolescents (21.59%) and adolescents in the region of origin (24.37%); the difference was statistically significant (*p* < 0.001; see Table 2 and Fig. 2). However, at W2, there was no significant difference in the prevalence of behavioral problems among the three groups. Table 3 presents the differences between W1 and W2 in the prevalence of behavioral problems, which was significant for the boys in the migrant adolescent group (χ^2^ = 6.49, *p* = 0.012^*,*^) and overall (χ^2^ = 7.35, *p* = 0.007), and was significantly lower at W2 than at W1. In the sensitivity analysis, imputing poor performance for missing data minimally affected the prevalence of behavioral problems (Supplementary Table S1), suggesting that the missing data did not reduce the representativeness of the sample.

**Table 2:** Prevalence of adolescents’ behavioral problems among three groups of adolescents in W1 and W2

	W1					W2				
	Migrant Adolescents	Host Adolescents	Adolescents in the region of origin			Migrant Adolescents	Host Adolescents	Adolescents in the region of origin		
Variable	*N* = 1544	*N* = 1718	*N* = 1543	χ^2^	*p*	*N* = 748	*N* = 553	*N* = 434	χ^2^	*p*
Male	214 (28.76)	170 (20.26)	182 (26.45)	16.47	< 0.001	80 (21.62)	60 (22.56)	50 (26.88)	2.00	0.369
Female	219 (27.38)	201 (22.87)	194 (22.69)	6.30	0.043	90 (23.81)	64 (22.30)	66 (26.61)	1.38	0.501
Total	433 (28.04)	371 (21.59)	376 (24.37)	18.30	< 0.001	170 (22.73)	124 (22.42)	116 (26.73)	3.09	0.213

**Table 3:** Longitudinal comparative analysis of the prevalence of behavioral problems among three groups of adolescents

Group			Constituent ratio	*χ*^*2*^	*p* value
Migrant Adolescents	Male	W1 (*N* = 744)	214 (28.76)	6.49	0.012
		W2 (*N* = 370)	80 (21.62)		
	Female	W1 (*N* = 800)	219 (27.38)	1.69	0.202
		W2 (*N* = 378)	90 (23.81)		
	Total	W1 (*N* = 1544)	433 (28.04)	7.35	0.007
		W2 (*N* = 748)	170 (22.73)		
Host Adolescents	Male	W1 (*N* = 839)	170 (20.26)	0.65	0.436
		W2 (*N* = 266)	60 (22.56)		
	Female	W1 (*N* = 879)	201 (22.87)	0.04	0.871
		W2 (*N* = 287)	64 (22.30)		
	Total	W1 (*N* = 1718)	371 (21.59)	0.17	0.679
		W2 (*N* = 553)	124 (22.42)		
Adolescents in the region of origin	Male	W1 (*N* = 700)	182 (26.45)	0.06	0.851
		W2 (*N* = 186)	50 (26.88)		
	Female	W1 (*N* = 843)	194 (22.69)	1.37	0.270
		W2 (*N* = 248)	66 (26.61)		
	Total	W1 (*N* = 1543)	376 (24.37)	1.01	0.315
		W2 (*N* = 434)	116 (26.73)		

### Multivariate results

As shown in Table 4, multivariable regression analysis indicated that, after adjusting for age and gender, behavioral problems at W1 were significantly associated with parents’ working outside the home as the main source of family income (*OR* = 1.42, 95% CI = 1.13–1.78), teacher-student relationships (OR = 0.97, 95% CI = 0.94–0.99), learning difficulties (OR = 1.04, 95% CI = 1.01–1.06), and parents who had no intention to move back to the original residence (OR = 0.70, 95% CI = 0.52–0.94). However, behavioral problems at W2 in the migrant adolescents were only significantly associated with improved living conditions (OR = 0.39–0.55, 95% CI = 0.25–0.84). For the host adolescents, behavioral problems at W1 were significantly associated with order and discipline (OR = 0.97, 95% CI = 0.95–1.00) and peer competition (OR = 1.03, 95% CI = 1.00–1.06). However, behavioral problems at W2 were significantly associated with parents’ lower education (OR = 1.64, 95% CI = 1.08–2.47), psychoticism (OR = 1.06, 95% CI = 1.00–1.13), and classmate relationships (OR = 0.94, 95% CI = 0.88–0.99; Supplementary Table S2). For the adolescents in the region of origin, only parents’ lower education had a statistically significant influence on behavioral problems at W2 (OR = 0.63, 95% CI = 0.40–0.99; Supplementary Table S3).

**Table 4:** Relationship between factors and behavioral problems in migrant adolescents based on univariate and multivariate logistic regressions

	W1(*N* = 1544)				W2 (*N* = 748)			
	Univariate regressions		Multivariate regression		Univariate regressions		Multivariate regression	
Factor	OR	95% CI	OR	95% CI	OR	95%CI	OR	95%CI
**Intrapersonal**								
Personality								
Extraversion	1.00	(0.98–1.01)	1.00	(0.97–1.03)	1.01	(0.97–1.06)	1.01	(0.96–1.05)
Neuroticism	1.00	(0.99–1.01)	1.00	(0.98–1.03)	0.99	(0.99–1.01)	0.99	(0.95–1.04)
Psychoticism	1.02	(0.99–1.04)	1.03	(0.99–1.07)	1.02	(0.99–1.04)	1.02	(0.96–1.09)
**Family**								
Parents’ highest education								
primary school	0.98	(0.78–1.23)	1.03	(0.82–1.29)	0.88	(0.55–1.42)	1.18	(0.74–1.91)
Middle school and above	1.00		1.00				1.00	
Family income sources								
Farming	1.00		1.00		1.00		1.00	
working outside the home	1.03^**^	(0.90–1.17)	1.42^**^	(1.13–1.78)	1.26	(0.82–1.93)	1.72	(0.96–3.06)
**Social environment**								
Teacher-student relationship	0.99	(0.98–1.02)	0.97^**^	(0.94–0.99)	1.00	(0.98–1.02)	1.01	(0.96–1.06)
Classmate Relations	0.99	(0.98–1.00)	1.00	(0.97–1.03)	1.01	(0.98–1.04)	1.00	(0.94–1.06)
Order and Discipline	0.99	(0.98–1.00)	0.99	(0.97–1.02)	1.01	(0.98–1.05)	1.03	(0.97–1.08)
Peer competition	1.01	(0.99–1.02)	0.99	(0.97–1.02)	1.00	(0.97–1.02)	1.00	(0.95–1.07)
Learning Burden	1.02	(1.01–1.04)	1.04^**^	(1.01–1.06)	0.98	(0.95–1.01)	0.96	(0.90–1.02)
**Ecological migration**								
Parents’ desire to move back to original residence								
No	1.03	(0.88–1.20)	0.70^*^	(0.52–0.94)	0.85	(0.56–1.30)	0.88	(0.46–1.67)
Occasionally	1.06	(0.89–1.26)	0.94	(0.71–1.24)	1.09	(0.67–1.78)	0.86	(0.48–1.55)
Often	1.00		1.00		1.00		1.00	
Living condition is better than before								
No	1.00		1.00		1.00		1.00	
Somewhat	0.92	(0.76–1.10)	1.04	(0.75–1.45)	0.41^**^	(0.27–0.64)	0.39^**^	(0.25–0.62)
Completely	1.00	(0.87–1.16)	1.20	(0.92–1.58)	0.60^**^	(0.40–0.89)	0.55^**^	(0.36–0.84)

* *p* < 0.05; ** *p* < 0.01

Multivariate regression adjusted for age and sex

## Discussion

This study makes an important contribution to the migration literature because it is the first to examine adolescent behavioral problems among ecological migrants who participated in China’s resettlement program. Our study has three important findings. First, at W1, from 2012 to 2014, the prevalence of behavioral problems among the migrant adolescents (28.04%) was significantly higher than among host adolescents (21.59%) and adolescents in the region of origin (24.37%). Second, the prevalence of behavioral problems among the migrant adolescents significantly decreased about 2 years later (22.73%) at W2 (2014–2017), and there was no longer a significant difference among the three groups of adolescents. This may suggest that behavioral problems related to leaving one’s place of origin and adapting to a new place diminish over time. However, this may have also been related to the fact that the migrant participants from W1 excluded from W2 (14–16 years old participants at W1) had significantly higher CBCL scores than migrant participants retained at W2, possibly indicating that migration has a differential impact on different age groups in the initial period. Thus, more research is needed with age-stratified adolescent groups to verify this finding. If the differential effects of age are verified, interventions should be created that target different age groups of migrant adolescents.

The present findings offer insights into the ways in which the migration experience influences adolescent behavior and adds to our contextual knowledge regarding adolescent behavioral change. Third, our findings clarify that parents’ thoughts and behaviors influence migrant adolescents’ behaviors. That is, having parents who work outside the home and harbor a desire to move back to the original residence both increased the risk of behavioral problems. However, a good relationship with teachers and other students seemed to have a protective effect at W1, although learning burdens seemed to increase behavioral problems.

At W1, behavioral problems were most prevalent in the migrant adolescent group, which supports the results of previous studies [34, 35]. Other studies have found that behavioral problems in immigrant children are significantly associated with an array of factors related to family, the social environment, and ecological migration [36–38]. Although the relocation and resettlement of ecological migrants was organized and implemented by the government, this policy is predominantly concerned with moving residents from an area with poor ecological carrying capacity to one with a better capacity in order to improve people’s lives. However, migration tends to be stressful, as adapting to a new environment and culture is always challenging [39]. Adolescent immigrants face isolation, acculturation-related stress, ethnic discrimination, and challenges related to community and school integration [40–42]. We found that parents working outside the home to generate family income seemed to be a risk factor regarding migrant adolescents’ behavioral problems at W1 and W2. One explanation for this finding is that parents who worked outside the home had less time to monitor and support their adolescents than did parents who farmed at home [36]. During the first 2 years after migrating, adolescents tend to adapt to their new environment and successfully pass through a period of psychological vulnerability. If they had been left in their resettlement destination by parents who left to work elsewhere, it would usually be difficult for such adolescents to obtain timely and sufficient support [43]. A previous study found that left-behind children had more behavioral problems than did their counterparts living with their parents [44–46].

We found that parents with no intention to move back to their original residence at W1 and who experienced improved living conditions at W2 were protective factors against adolescents developing behavioral problems. The Ecological Migration Project has been a key environmental policy in which thousands of people have participated, especially in the northern areas of China. Indigenous people who have long lived in the ecologically fragile mountainous southern areas that receive scant rainfall and have poorly nourished land, had a per capita gross domestic product (GDP) of less than 1500 US dollars in 2012, whereas those in the more developed northern plains region located along the Yellow River had a per capita GDP of over 5000 US dollars in 2012, as well as options for convenient transportation and an improved living environment overall. Migration is often motivated by economics, education, and environment, among other factors. Host residents also tend to have higher socioeconomic status than resident who remain in the region of origin, which may benefit migrants. At the early stage of migration, parents’ identification with the new environment in the migrant area increases children’s sense of identity and integration into the host destination. The changes in parents’ livelihood after resettlement might improve the family’s living conditions through additional material and family support, alleviate the negative psychological effects of migration, and serve as an important protective factor against adolescent behavioral problems [47]. Examples include the availability of clean water sources, abundant food options, balanced nutrition, warmer and safer heating options, and clean cooking methods. Community and healthcare services are also higher quality in the northern region [48], and there are various entertainment opportunities, such as sports facilities and multimedia classrooms.

Research has recently focused on the creation of student-teacher relationships as a positive source of protection and as a source of negative risk regarding a variety of student outcomes [49]. We explored student-teacher relationships relative to adolescent behavioral problems and found that better relationships with teachers correlated with fewer behavioral problems both at W1 and 2 years later at W2. Our results are consistent with previous findings on this relationship [50–52]. It may be that students consider relationships with teachers to be as important as family ties and support [53]. Several human development theories emphasize the role of student-teacher relationships in student development, such as attachment theory [54], socialization theory, interpersonal relationship theory, development system theory, and social motivation theory [55]. These theories emphasize different aspects of student-teacher interaction, but they all agree that emotional support, connectivity, intimacy, and sensitivity are key determinants of the relationship’s quality. Healthy and strong interpersonal relationships also foster healthy socio-emotional development and well-being [56, 57]. Further, they might steer adolescents with high initial levels of problem behaviors away from a trajectory of increasing problems throughout adolescence. Educators and schools could provide awareness and training about migration in addition to support for teachers to develop positive relationships with their students, particularly when they are teaching migrants.

We found that learning burdens were related to behavioral problems at W1, and we acknowledge that the expectations and quality of material taught at the new schools are usually higher than in the original schools. However, we found that the migrant adolescent group gradually adapted to their new schools, as the influence of learning burden on behavioral problems at W1 became statistically non-significant at W2. From the perspective of the ecological environment, multi-level interventions could be implemented at schools for adolescents and their families in the early stages of immigration to help strengthen their learning skills [58, 59]. These efforts could be implemented throughout primary, middle, and high school to focus on specific stages of education and child development, and be tailored to subsets of migrant children.

Despite our study’s valuable results and advances, a few limitations should be noted in assessing its contribution and planning future research. First, there are currently approximately 430,000 ecological migrant adolescents in the mountainous southern areas of Ningxia, and our sample size was small relative to this number. However, we included cases from all the settlements, so the results may, nevertheless, be representative. Our study was conducted in one Chinese province, and a broader sampling strategy would be helpful to learn more about adolescent behaviors. Second, there are considerable socioeconomic differences among migrant families; thus, more research is required concerning family finances and family dynamics. Third, the CBCL is generally considered to be comprehensive and accurate in assessing youth behavioral problems; however, our results may have been affected by the education level of the parents in this study. Although researchers entered the household to guide parents in filling out the questionnaire during data collection, the education level of this part of the population was relatively low, which led to some data being inadmissible due to missing responses. Fourth, we excluded participants diagnosed with any mental disorders, which may have inadvertently excluded adolescents with more severe behavioral problems. However, mental health conditions that occurred before migration could have acted as confounding factors for the adolescent behavioral problems related to migration. Future research should consider exploring the impact of migration on adolescents with pre-existing mental health problems. Fifth, attrition bias presents a concern for the generalizability of our results. While this is an inherent limitation in all prospective research when working with mobile populations, attrition analysis showed that the two samples did not differ significantly on CBCL scores. However, we also excluded participants from W2 if they were 17 years or older after W1; thus, future research could focus on adolescents over the age of 16. Finally, some participants did not fill in their children’s names during the follow-up, resulting in an incomplete match with the first dataset, which limited the longitudinal data analysis.

## Conclusion

Although it has limitations, this is the first study to examine adolescent behavioral problems in a sample of ecological migrants participating in China’s Ecological Migration Project. We found that behavioral problems were significantly higher among migrant adolescents compared with host adolescents or adolescents in the region of origin; however, after 2 years, the behavioral problems significantly decreased. Parents working outside the home, parents having no intention to move back to their original residence, and learning burdens increased the risk of behavioral problems. Conversely, improved living conditions and strong student-teacher relationships were protective factors. Our findings underscore the importance of comprehensively considering the factors that influence migrant children’s behaviors. The results suggest that policymakers should continue to develop strategies to improve the lives of migrants. To do so, it is necessary to recognize the most important risk and protective factors for ecological migrant children.

## Supplementary information

**Additional file 1: Supplementary Table 1**. Means and standard deviations of behavioral problems among three groups of adolescents in W2. **Supplementary Table 2**. Relationship between influencing factors and behavior problems in host adolescents base on univariate and multivariate logistic regressions. **Supplementary Table 3**. Relationship between influencing factors and behavior problems in adolescents in the region of origin base on univariate and multivariate logistic regressions.

## Data Availability

The datasets used and analyzed during the current study are available from the corresponding author on reasonable request.

## Abbreviations

CBCL: Child Behavior Checklist
EPQ: Eysenck Personality Questionnaire
MCI: My Class Inventory Questionnaire
OR: Odds Ratio
CI: Confidence Interval
