# Supplementary material for: Migrant adolescents’ behavioral problems compared to host adolescents and adolescents in their region of origin: a longitudinal study
Source: BMC Psychiatry. 2020 Sep 29;20:472. doi: 10.1186/s12888-020-02872-x (PMC7526236; doi:10.1186/s12888-020-02872-x)
Supplement: Supplementary file 1 — Additional file 1: Supplementary Table 1. Means and standard deviations of behavioral problems among three groups of adolescents in W2. Supplementary Table 2. Relationship between influencing factors and behavior problems in host adolescents base on univariate and multivariate logistic regressions. Supplementary Table 3. Relationship between influencing factors and behavior problems in adolescents in the region of origin base on univariate and multivariate logistic regressions. [file 12888_2020_2872_MOESM1_ESM.docx]

Supplementary table 1：Means and standard deviations of behavioral problems among three groups of adolescents in W2

| Group | N | Mean (SD) | F | *P* |
| --- | --- | --- | --- | --- |
| Migrant Adolescents | 1544 | 24.47（32.44） | 1.298 | 0.273 |
| Host Adolescents | 1718 | 22.85（28.67） |  |  |
| Adolescents in the region of origin | 1543 | 23.92（26.43） |  |  |

Supplementary table 2：Relationship between Influencing factors and behavior problems in host adolescents base on univariate and multivariate logistic regressions

|  | W1（N=1718） | | | |  | W2(N=553) | | | | | |
| --- | --- | --- | --- | --- | --- | --- | --- | --- | --- | --- | --- |
|  | Univariate regressions | | Multivariate regression | |  | Univariate regressions | | | Multivariate regression | | |
| Factor | OR | 95% CI | OR | 95% CI |  | OR | | 95%CI | OR | | 95%CI |
| **Intrapersonal** |  |  |  |  |  |  |  | |  |  | |
| Personality |  |  |  |  |  |  |  | |  |  | |
| Extraversion | 1.01 | (0.98-1.03) | 1.00 | (0.98-1.03) |  | 0.99 | (0.95-1.03) | | 1.00 | (0.95-1.06) | |
| Neuroticism | 1.00 | (0.98-1.02) | 1.00 | (0.98-1.03) |  | 0.98 | (0.94-1.01) | | 0.95 | (0.91-1.00) | |
| Psychoticism | 1.03 | (0.99-1.07) | 1.04^*^ | (1.00-1.09) |  | 1.02 | (0.97-1.08) | | 1.06^*^ | (1.00-1.13) | |
| **Family** |  |  |  |  |  |  |  | |  |  | |
| Parents’ highest education |  |  |  |  |  |  |  | |  |  | |
| primary school | 1.17 | (0.92-1.49) | 1.14 | (0.87-1.47) |  | 1.54^*^ | (1.03-2.30) | | 1.64^*^ | (1.08-2.47) | |
| Middle school and above | 1.00 |  | 1.00 |  |  | 1.00 |  | | 1.00 |  | |
| Family income sources |  |  |  |  |  |  |  | |  |  | |
| Farming | 1.00 |  | 1.00 |  |  | 1.00 |  | | 1.00 |  | |
| working outside the home | 0.89 | (0.71-1.12) | 0.95 | (0.67-1.36) |  | 1.16 | (0.78-1.72) | | 1.12 | (0.74-1.70) | |
| **Social environment** |  |  |  |  |  |  |  | |  |  | |
| Teacher-student relationship | 1.01 | (0.99-1.02) | 1.01 | (0.99-1.03) |  | 0.98 | (0.95-1.01) | | 1.02 | (0.97-1.07) | |
| Classmate Relations | 0.99 | (0.98-1.01) | 0.99 | (0.96-1.02) |  | 0.96^*^ | (0.93-1.00) | | 0.94^*^ | (0.88-0.99) | |
| Order and Discipline | 0.99 | (0.97-1.00) | 0.97^*^ | (0.95-1.00) |  | 0.99 | (0.96-1.03) | | 1.03 | (0.98-1.07) | |
| Peer competition | 1.02^*^ | (1.00-1.05) | 1.03^*^ | (1.00-1.06) |  | 0.98 | (0.95-1.01) | | 0.97 | (0.93-1.01) | |
| Learning Burden | 1.02 | (1.00-1.05) | 1.02 | (0.99-1.04) |  | 1.03 | (0.99-1.08) | | 1.04 | (1.00-1.09) | |

* p＜0.05; ** p＜0.01;

Multivariate regression adjusted for age and sex.

Supplementary table 3：Relationship between Influencing factors and behavior problems in adolescents in the region of origin base on univariate and multivariate logistic regressions

|  | W1（N=1543） | | | |  | W2(N=434) | | | |
| --- | --- | --- | --- | --- | --- | --- | --- | --- | --- |
|  | Univariate regressions | | Multivariate regression | |  | Univariate regressions | | Multivariate regression | |
| Factor | OR | 95% CI | OR | 95% CI |  | OR | 95%CI | OR | 95%CI |
| **Intrapersonal** |  |  |  |  |  |  |  |  |  |
| Personality |  |  |  |  |  |  |  |  |  |
| Extraversion | 1.00 | (0.97-1.02) | 0.99 | (0.96-1.02) |  | 1.04 | (0.99-1.10) | 1.05 | (1.00-1.11) |
| Neuroticism | 1.02 | (1.00-1.04) | 1.02 | (1.00-1.05) |  | 0.98 | (0.94-1.03) | 0.98 | (0.94-1.04) |
| Psychoticism | 1.02 | (0.98-1.05) | 1.00 | (0.97-1.04) |  | 1.01 | (0.94-1.09) | 1.03 | (0.95-1.11) |
| **Family** |  |  |  |  |  |  |  |  |  |
| Parents’ highest education |  |  |  |  |  |  |  |  |  |
| primary school | 0.98 | (0.77-1.24) | 0.96 | (0.76-1.22) |  | 0.65 | (0.42-1.01) | 0.63 | (0.40-1.00) |
| Middle school and above | 1.00 |  | 1.00 |  |  | 1.00 |  | 1.00 |  |
| Family income sources |  |  |  |  |  |  |  |  |  |
| Farming | 1.00 |  | 1.00 |  |  | 1.00 |  | 1.00 |  |
| working outside the home | 0.94 | (0.74-1.19) | 0.94 | (0.73-1.22) |  | 1.07 | (0.64-1.78) | 0.67 | (0.35-1.28) |
| **Social environment** |  |  |  |  |  |  |  |  |  |
| Teacher-student relationship | 1.01 | (0.99-1.02) | 1.01 | (0.98-1.03) |  | 0.99 | (0.96-1.02) | 0.98 | (0.94-1.02) |
| Classmate Relations | 1.00 | (0.98-1.03) | 0.99 | (0.96-1.02) |  | 0.99 | (0.94-1.03) | 1.01 | (0.95-1.08) |
| Order and Discipline | 1.01 | (0.99-1.04) | 1.01 | (0.98-1.04) |  | 0.98 | (0.94-1.02) | 0.95 | (0.90-1.01) |
| Peer competition | 1.01 | (0.99-1.03) | 1.00 | (0.98-1.03) |  | 1.01 | (0.97-1.06) | 1.02 | (0.96-1.09) |
| Learning Burden | 1.01 | (0.99-1.04) | 1.01 | (0.99-1.04) |  | 1.02 | (0.97-1.07) | 1.04 | (0.97-1.12) |

* p＜0.05; ** p＜0.01;

Multivariate regression adjusted for age and sex.
